# Potential of LC Coupled to Fluorescence Detection in Food Metabolomics: Determination of Phenolic Compounds in Virgin Olive Oil

**DOI:** 10.3390/ijms17101627

**Published:** 2016-09-24

**Authors:** Romina P. Monasterio, Lucía Olmo-García, Aadil Bajoub, Alberto Fernández-Gutiérrez, Alegría Carrasco-Pancorbo

**Affiliations:** 1 Instituto de Biología Agrícola de Mendoza (IBAM), CONICET, Alt. Brown 500, Chacras de Coria, 5505 Mendoza, Argentina; rmonasterio@mendoza-conicet.gob.ar; 2 Department of Analytical Chemistry, Faculty of Sciences, University of Granada, Ave. Fuentenueva s/n, E-18071 Granada, Spain; luciaolmo@ugr.es (L.O.-G.); aliam80@hotmail.com (A.B.); albertof@ugr.es (A.F.-G.)

**Keywords:** fluorescence detection, olive oil, phenolic compounds, secoiridoids, food metabolomics

## Abstract

A powerful chromatographic method coupled to a fluorescence detector was developed to determine the phenolic compounds present in virgin olive oil (VOO), with the aim to propose an appropriate alternative to liquid chromatography-mass spectrometry. An excitation wavelength of 285 nm was selected and four different emission wavelengths (316, 328, 350 and 450 nm) were simultaneously recorded, working therefore on “multi-emission” detection mode. With the use of commercially available standards and other standards obtained by semipreparative high performance liquid chromatography, it was possible to identify simple phenols, lignans, several complex phenols, and other phenolic compounds present in the matrix under study. A total of 26 phenolic compounds belonging to different chemical families were identified (23 of them were susceptible of being quantified). The proposed methodology provided detection and quantification limits within the ranges of 0.004–7.143 μg·mL^−1^ and 0.013–23.810 μg·mL^−1^, respectively. As far as the repeatability is concerned, the relative standard deviation values were below 0.43% for retention time, and 9.05% for peak area. The developed methodology was applied for the determination of phenolic compounds in ten VOOs, both monovarietals and blends. Secoiridoids were the most abundant fraction in all the samples, followed by simple phenolic alcohols, lignans, flavonoids, and phenolic acids (being the abundance order of the latter chemical classes logically depending on the variety and origin of the VOOs).

## 1. Introduction

Virgin olive oil (VOO), the juice of the olive obtained by pressing, is one of the few oils that are consumed without any further refining process. For that reason, it contains several bioactive molecules (vitamins, carotenoids, tocopherols, phenolic compounds, and some other natural antioxidants), which may act, by different mechanisms, as an effective defense against reactive oxygen substances [1]. Among its several minor constituents, phenolic compounds attract considerable attention because of their connection with some healthy benefits, including the prevention of chronic diseases such as cancer, obesity, diabetes, or coronary diseases [2,3]. Moreover, they contribute to the stability of VOO against auto-oxidation and have an important role in its organoleptic properties (bitterness, pungency, and astringency) [4,5]. These metabolites are also important from a commercial point of view, since the phenolic profile of a VOO can be a very useful distinctive feature to assure, for instance, its geographical origin [6,7] or authenticate its variety [8,9], which are two criteria considered by the international protection labels of geographical indications (e.g., Protected Designations of Origin and Protected Geographic Indications). Bearing in mind all the reasons just given, the importance of this group of secondary metabolites seems undeniable; indeed, over the last decade, these compounds have been considered as relevant targets in the field of food metabolomics.

In addition, declaring the phenolic content of a VOO in its label can represent a strategy for attracting consumer’s attention, since international organisms such as the European Food Safety Authority (EFSA) have approved the use of health claims concerning olive oil phenolic compounds. Their scientific panel has recognized a relationship between the consumption of olive oil phenolic compounds and the protection of LDL particles from oxidative damage [10]. The European Union (EU), as the world leading olive oil producer, established in 2012 a list of permitted health claims made on foods and restricted the use of the just mentioned claim to olive oils which contain at least 5 mg of hydroxytyrosol (HTY) and its derivatives (e.g., oleuropein complex and tyrosol (TY)) per 20 g of olive oil [11].

Nevertheless, there are some issues that are depriving the producers and consumers of the benefits derived from this regulation. As stated in a very interesting recent report [12], two main problems should be addressed: (a) the lack of clarity in terminology (“olive oil” and “polyphenols” are terms which are sometimes not properly used; even the EU conditions of use of the claim are not clearly formulated); and (b) the absence of a suitable analytical protocol for the determination of the bioactive compounds behind the claim. A more comprehensive discussion concerning the first specified problem is perhaps beyond the scope this manuscript. With regard to the second one, different approaches have been used so far: determination of the total content on phenolic compounds (colorimetric methods, using the Folin–Ciocalteu reagent); assessment of total HTY and TY content by liquid (LC) or gas chromatography after appropriate sample preparation (hydrolysis of bound forms); application of nuclear magnetic resonance procedures; profiling and individual determination of the phenolic compounds by using powerful chromatographic methodologies, etc. LC methods can be pointed out among the preferred ones, because they allow the individual determination of phenolic compounds and are simple, repeatable and easily adapted to routine laboratories. The lack of commercially available pure standards (only accessible for some of the phenolic compounds found in VOO) is a serious problem, since this fraction is considerably complex. It is composed by a heterogeneous mixture of compounds belonging to different families with varying chemical structures (simple phenolic alcohols, phenolic acids, flavonoids, lignans, secoiridoids, etc.). At least 32 structurally distinct phenolic compounds have been identified in this matrix [1].

Although LC has been used coupled to different detectors, mass spectrometry (MS) is becoming almost mandatory to overcome the above-mentioned issue (absence of appropriate pure standards) [1,13]. Unfortunately, this detector is not always available in routine laboratories due to its high acquisition and maintenance costs. Some other less expensive detection techniques such as UV absorption or fluorescence (FL) [1,14] could be, therefore, good alternatives. FL has been used considerably less than UV detection, although it shows, in some cases, higher selectivity and sensitivity, so it could be offered as a robust and reliable alternative to MS detection systems.

A limited number of LC-FL methods in the field of olive oil analysis have been published, and they have been mainly focused on the determination of few compounds belonging to some particular families such as lignans [15,16], phenyl alcohols and phenolic acids [17,18,19,20] or phenyl alcohols and secoiridoid derivatives [21,22,23,24]. In fact, the use of additional detectors was imperative in most of the cases to determine a major number of phenolic compounds. Nevertheless, the molecular structure of phenolic compounds makes them potentially detectable by a fluorescence detector (FLD), as they are natural fluorophores (that typically contain aromatic groups, or combined π bonds) which absorb energy of a specific wavelength and emit it at another particular higher wavelength (with less energy), depending on their structure and chemical environment [25,26,27].

The aim of the present work has been to develop a LC-FLD method for the identification and quantification of a noteworthy number of phenolic compounds in VOO samples belonging to different chemical classes. After a deep evaluation of the spectral behavior of the compounds under study (using commercial standards and standards obtained by semipreparative high performance liquid chromatography (HPLC)), a multi-emission wavelengths strategy was pointed out as optimum, and it gave us the possibility of characterizing simple phenols, lignans, several complex phenols and other phenolic compounds within a single run. This is the first method implying the use of a FLD, which is able to achieve qualitative and quantitative information of about 23 phenolic compounds.

## 2. Results and Discussion

### 2.1. Preliminary FLD Study and Compounds Identification

The FLD method development obviously began with a literature review, trying to identify a promising starting point regarding excitation and emission wavelengths. Apart from the interesting information included in previously published manuscripts [2,3,4,5], we took into account the fact that an absorption spectrum is a good starting point for selecting the excitation wavelength of an analyte, because the spectrum indicates which energy is absorbed to excite an electron to a higher quantum state. The absorption maximum, quite often, is pretty similar to the excitation maximum, so 240 and 280 nm were selected in this case in a first stage of our study, as excitation wavelengths to start the deep and rigorous fluorescence characterization of the substances under evaluation. In a subsequent step, zero order emission mode was used (fixing the excitation first at 240 nm and, then, at 280 nm). This kind of emission mode sets the monochromator so that all light emitted from the sample will be reflected onto the detector regardless of the emission spectrum. In such a way, we collected relevant information about the maximum emission wavelengths (325, 360 and 450 nm), afterwards fixing those values to carry out zero order-excitation spectra, for re-evaluating therefore the suitability of the initially selected excitation features (maximum λ_exc_). In this sort of excitation mode, the full spectrum of light from the Xenon lamp illuminates the flow cell and each compound can absorb its distinctive wavelength of light and then emit maximum fluorescence.

Bearing in mind the complexity of the phenolic fraction under study and the number of analytes which composed the extracts, the criteria considered to select the optimum excitation and emission wavelengths were: (1) to have the possibility of determining in VOO samples as many compounds as possible (belonging to different chemical classes) within a single run; (2) to increase, if possible, the selectivity by using proper wavelengths in multi-excitation or multi-emission conditions; and (3) to favor the achievement of a method with the best possible sensitivity and enhanced analytical parameters.

After the preliminary studies and trying to sweep the whole excitation range, four zero order-emission spectra were recorded, fixing 210, 236, 285 and 300 nm as λ_exc_. As a result of their evaluation, 285 nm seemed to be the most adequate excitation wavelength to start with (further details in Section 2.2). Setting this value, a 3D plot was acquired with zero order emission; this kind of plot displays a three dimensional image of the data file including spectra. The x-axis represents the retention time, the y-axis the wavelength and the z-axis the emission signal of the sample. The 3D plot shows peaks belonging to every fluorescent compound in the sample, which appear at different depth in the z-axis depending on its emission wavelength. It gives to the analyst the chance to explore the spectral landscape from a complete run, since by tilting, swiveling or turning the graphic it is possible to reveal hidden analyte characteristics in complex mixtures.

**Figure 1:**
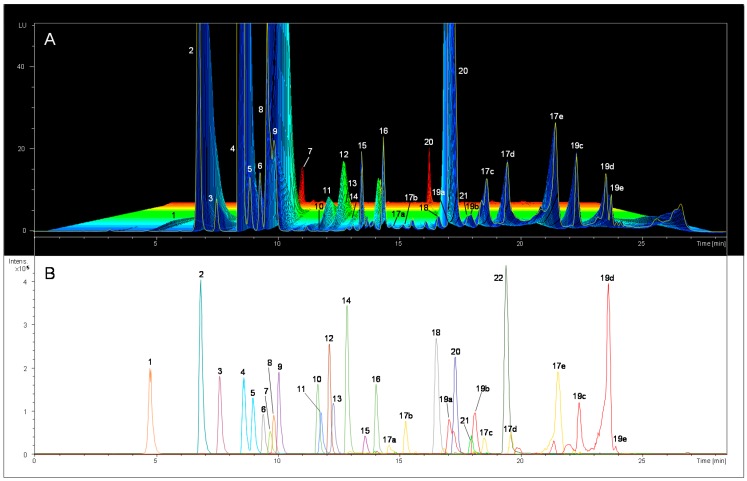
(**A**) 3D-plot of a fortified extra-VOO extract with eighteen phenolic compounds at a final concentration level of 10 μg·mL^−1^, when excitation wavelength is set at 285 nm and the zero order emission spectra is recorded; (**B**) Extracted ion chromatograms (EICs) of the known phenolic compounds for the same fortified extract as in (**A**), obtained in electrospray ionization–ion trap MS (ESI-IT MS) detector (using negative ionization mode). Peak identification numbers: (1) Gallic acid (Gal); (2) HTY; (3) 3,4-dihydroxyphenylacetic acid (DOPAC) (used as internal standard (IS)); (4) TY; (5) 4-hydroxybenzoic acid (4-HBA); (6) 4-hydroxyphenylacetic (4-HPA); (7) vanillic acid (Van); (8) syringic acid (Syr); (9) homovanillic acid (Hmvan); (10) *p*-coumaric acid (*p*-Cou); (11) vanillin (Val); (12) sinapic acid (Sin); (13) ferulic acid (Fer); (14) *m*-coumaric acid (*m*-Cou); (15) oleuropein (Ole); (16) *o*-coumaric acid (*o*-Cou); (17) oleuropein aglycon (OleAgly) and isomers; (18) luteolin (Lut); (19) ligstroside aglycon (LigAgly) and isomers; (20) (+)-pinoresinol (Pin); (21) acetoxypinoresinol (AcPin); and (22) Apig. As far as the different isomers of OleAgly and LigAgly are concerned, they are identified by adding a letter (a–e) to the number assigned for the main isomer.

At this point and with the aim of achieving the identity of as many peaks as possible within the profiles, before keep going with the FLD optimization, we used different kind of standards (commercially available standards and the previously isolated phenolic compounds by semi-preparative HPLC), as well as a MS detector coupled to the HPLC-FLD instrument. Therefore, the identities of the phenolic compounds were established by comparison of these 3D plots with MS chromatograms for standard mix solutions, VOO samples and fortified samples (taking into account the retention time of each peak). Figure 1 shows, in the upper part, a 3D FL chromatogram (λ_exc_ = 285 nm and zero order emission) of a fortified extra-VOO extract; and in the lower part, the equivalent MS chromatogram (in both cases, peaks are identified with the pertinent numbers). In this figure, apigenin (Api) peak can only be found in the MS chromatogram (it does not appear in FLD 3D plot) because this compound does not fluoresce with enough intensity (regarding fluorescence efficiency, the same was observed for the isolated standards of elenolic acid (EA) and decarboxymethyl oleuropein aglycon (DLA) whose FL signal was undetectable).

Supplementary Table S1 shows the retention time, molecular formula, chemical structure, the family of compounds at which they belong to, and the optimum spectral parameters (obtained in methanol) of the phenolic compounds which could be quantified by our method. The table also includes the FL emission channel at which each compound will be more satisfactorily detected (this point is extensively explained in the following section). Apart from the compounds identified in the sample in Figure 1, three more compounds can be seen in the Supplementary Table S1: oxidized hydroxytyrosol (OxHTY), hydroxytyrosol acetate (AcHTY) and decarboxymethyl oleuropein aglycon (DOA). In total, 23 compounds (and seven isomers) are presented in this table, which were further quantified in the samples. In addition to the compounds that were quantified, hydroxy elenolic acid, syringaresinol and 10-hydroxy oleuropein aglycone, which have not been previously reported using FLD, were found in some of the samples.

### 2.2. Optimization of Detection Conditions

After the preliminary evaluation of the FL conditions and achieving the identification of an important number of analytes within the profiles, we got ready to face the final optimization of the conditions for fluorescence detection (obviously under the optimum LC conditions), as well as to select the most appropriate fluorescence mode (segments with time programming, multi-channels, etc.). For that purpose, the standard mix solution, the isolated phenolic compounds, the quality control (QC) and fortified sample extracts were used.

As stated in the previous section, the establishment of a proper starting value for excitation wavelength was the first stage of the detection method development; 285 nm was the one that generated a chromatogram with the major number of peaks. The zero order emission spectrum obtained while setting excitation wavelength at 285 nm was studied in depth in order to find the emission maximum for each phenolic compound (apart from those logically found in the preliminary study). As many zero order excitation and emission spectra as necessary (selecting different wavelengths and conditions) were done in order to identify the maximum λ_exc_ and λ_em_ for each compound. The resulting values are included in Supplementary Table S1.

After corroborating the excitation and emission wavelengths that produced the most promising FL chromatograms, it was required to choose the most appropriate operational mode. Our FLD was able to work in the following modes: 3D spectral mode (for the rapid online acquisition of full excitation and emission spectra); time programming of spectral (where a timetable is created by the analyst defining different segments where diverse FL conditions are applied (excitation and emission wavelengths, PMT gain, bandwidth, attenuation, etc.)); and multi-emission (where an excitation wavelength is fixed and different emission wavelengths are simultaneously recorded) or multi-excitation mode. The potential of the first mentioned mode (3D spectral mode) was already used in previous stages of the current study. The usefulness of the second one—the creation of segments with different emission wavelengths during the run—was investigated in order to adequate the detection conditions in each segment for the compounds eluting at those retention times and, therefore, to be capable of determining all the phenolic compounds in only one chromatogram; in other words, defining different analytical windows within the same chromatographic trace. When this mode was selected, it was necessary to overcome several difficulties: (1) great number of segments needed (at least 20) to assure that each phenolic compound was determined by using the optimum wavelengths; (2) the fact that the change of the conditions between two segments was sometimes drastic and it caused the loss of information when the peaks next to the edge of the segment were too close (the instrument requires some time to change the operational parameters); and (3) the remarkable fluctuations of the baseline when using different FL conditions, fact which made the obtained chromatograms very difficult to process.

The third operational mode, multi-channel (in particular, multi-emission mode), was then used. A wavelength of 285 nm had been clearly pointed out as optimum excitation wavelength. Considering the results included in Supplementary Table S1 regarding the maximum λ_em_ and trying to look for a compromise solution limiting the number of selected wavelengths for this stage of the optimization, four λ_em_ were chosen: 316, 328, 350 and 450 nm. These values allowed the determination of an important number of analytes with an adequate intensity (at very close emission wavelengths to the optimum found for each substance) with the best possible selectivity. In this mode the FLD was able to monitor phenols separation simultaneously in four channels with different emission wavelengths (while maintaining constant the excitation wavelength). In addition, when the emission of an analyte saturates the detector, its quantification can be made in a less sensitive wavelength. This is an important advantage to determine analytes whose concentration range varies a lot depending on the selected sample (as happens with phenolic compounds extracted from different VOOs).

**Figure 2:**
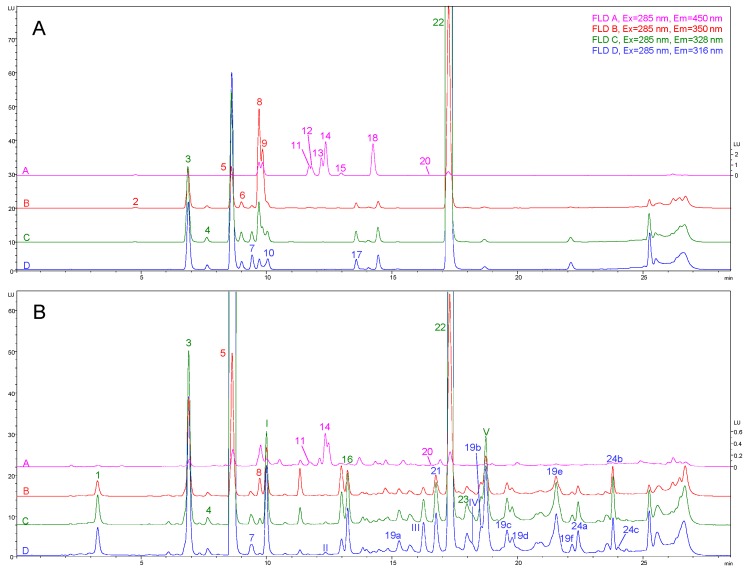
Chromatograms of: the standard mix with eighteen phenolic compounds at a concentration level of 10 μg·mL^−1^ (**A**); and a sample of olive oil from Picual variety (**B**). Peak identification numbers: (1) OxHTY; (2) Gal; (3) HTY; (4) DOPAC (IS); (5) TY; (6) 4-HBA; (7) 4-HPA; (8) Van; (9) Syr; (10) Hmvan; (11) *p*-Cou; (12) Val; (13) Sin; (14) Fer; (15) *m*-Cou; (16) AcHTY; (17) Ole; (18) *o*-Cou; (19) OleAgly isomers (20) Lut; (21) DOA; (22) Pin; (23) AcPin; and (24) LigAgly isomers. As far as the different isomers of OleAgly and LigAgly are concerned, they are identified by adding a letter (a–f) to the number assigned for the main isomer. Peak identification codes for those compounds which were not quantified: (I) hydroxytyrosol mass isomer; (II) hydroxy elenolic acid; (III) syringaresinol; (IV) 10-hydroxy oleuropein aglycone; and (V) pinoresinol mass isomer.

Figure 2 shows the chromatograms of a standard mix with 18 phenolic compounds and a sample of olive oil from Picual variety; four traces are presented, corresponding to the four emission channels in the FLD. Although some of the analytes under study could be detected by using different chromatographic traces, they have been just numbered in the most adequate one (which is also presented in Supplementary Table S1). Nevertheless, Ole and TY are numbered in the traces that have been finally used for their quantification (traces achieved by using a λ_em_ value that does not match with their maxima λ_em_ shown in Supplementary Table S1). Ole was detected at 316 nm instead of 328 nm because all secoiridoid derivatives, whose optimum λ_em_ was found at 316 nm, were quantified with respect to its calibration curve and it seemed to be more adequate to detect all of them at the same λ_em_. With regard to TY, its optimum λ_em_ was found at 316 nm but, as signal saturation at low concentration levels was observed, it was more favourable to quantify it at 350 nm. In the extract of Picual olive oil, 19 compounds (with 7 isomers; 5 of OleAgly and 2 of LigAgly) could be detected; 14 of them could be quantified if found within the linear dynamic range of the method (in terms of their own standards (if available) or using for that purpose another structure-related compound). The list of the compounds identified in the caption to figure by using Roman numbers, includes three substances which have not been previously determined by FLD (hydroxy elenolic acid, syringaresinol and 10-hydroxy oleuropein aglycone) and two mass isomers of HTY and Pin, respectively, which were found at min 10.0 and 18.7 but whose identity has not been confirmed.

Concerning the fluorescence behavior of the different compounds, it seems convenient to mention that cyclic molecules may have activators, such as –OH and –OR groups substituted in *o-* or/and *p-*positions, which produce an enhanced fluorescence, and deactivators such as –COOH, –COOR, –CHO and –COR groups substituted in *m-* positions, which induce a decrease of the fluorescence [28]. Therefore, in general, phenyl alcohols (OxHTY, HTY, TY and AcHTY) and lignans (Pin and AcPin) which have an activator group in *p-*position, are very susceptible to be detected by FL, producing peaks of considerable intensity within the profiles; and secoiridoid derivatives (with a phenyl alcohol group within their structure) can be also properly detected. A lower relative response is observed in those structures that contain a dialdehydic group in the open elenolic acid ring, since minor planarity of the molecule gives reduced fluorescence intensity [22]. Flavonoids also showed a very low fluorescence intensity (Api was not detected even at high concentrations), but this fact should not be considered as an issue or a factor with detrimental effect on the potential of the method, since it is well known that these compounds can be easily quantified at 330 nm with a UV detector (we indeed had diode-array detector (DAD) connected in series). The spectral characteristics of the phenolic acids included in our standard mix logically depends on their structure, so we could group these acids in two main classes: compounds with a benzoic-like structure (Gal, 4-HBA, Van, and Syr) whose optimum λ_em_ is 350 nm, and compounds with a hydroxycinnamic-like structure (*p*-Cou, Sin, Fer, *m*-Cou, and *o*-Cou) whose optimum λ_em_ is 450 nm. Nevertheless, 4-HPA which has a different kind of structure and Hmvan which is one exception with benzoic-like structure, have their optimum λ_em_ at 316 nm.

After deciding to maintain four emission channels, the very last step in the FL conditions optimization consisted of trying to maximize the fluorescent signals achieved. In this regard, we can stand out that an increase of one unit in the PMT gain parameter doubled the signal of the analytes, but also affected to the noise and declined the signal to noise ratio by more than a 30%. A PMT of 10 was the value finally selected for this parameter.

### 2.3. Method Validation

Validation studies were carried out to check the analytical performance of the developed method. Both the standard mixture containing the 18 phenolic compounds and the QC sample (serially diluted or fortified at different concentration levels) were used at this stage of the study. The calculated analytical parameters of the method are summarized in Table 1 and Table 2.

Method accuracy was assessed in terms of precision and trueness. The first one was evaluated by means of the *intra*-day and *inter*-day repeatability studies, by calculating the relative standard deviation (RSD) of the retention time and peak area of the analytes under study. The *intra*-day precision was expressed as the RSD obtained for five injections of the QC carried out within the same sequence and its values were below 0.39% (for retention time) and 9.05% (for peak area), for Gal and Hmvan, respectively. The *inter*-day precision was the RSD of five replicated injections of the QC belonging to five different sequences carried out over five days. Its values were below 0.99% (for retention time) and 11.98% (for peak area), for Pin and Hmvan, respectively. The trueness was expressed as recovery (%), which was calculated by analyzing the same sample extracted before and after the standard addition. Three concentration levels (low, intermediate and high) of the pure standards (within their linear range) were tested and resulting recovery values were found between 80.55% and 112.28%, for Sin and Ole, respectively.

Matrix effect was also evaluated in order to assess whether interferences may affect the spectral behavior of the phenolic compounds and hinder the proper quantification of the analytes under study. To do it so, a standard addition calibration curve was prepared by fortifying the QC with the standard mix at different concentration levels (within the range from 0.5 to 250 mg·L^−1^). Then, its slope was compared with the one of the external calibration curve, and the slope ratio (slope of the calibration curve in matrix/slope of the external calibration curve) was calculated for each analyte. If matrix does not affect the response of the phenolic compound being studied, the ratio between both slopes (b_matrix_/b_solvent_) should be between 0.80 and 1.20 [29]. Depending on the value of this ratio, different matrix effects could be observed: a value lower than 0.80 shows matrix suppression effect, whilst a value higher than 1.2 demonstrates matrix enhancement. As the values for the slopes ratio were found between 0.86 and 1.16 (for TY and Lut, respectively), the very low matrix effect observed made the external calibration appropriate for quantifying the phenolic compounds under study.

The linearity of the proposed method was checked by establishing solvent-based standard calibration curves within the range of 0.5 to 250 mg·L^−1^. A linear regression using the least-squares method was performed, and the peaks areas of each analyte (injected in triplicate) were plotted as a function of its concentration (eleven levels of concentration were tested). The responses properly fitted to a straight line with *R^2^* values between 0.9861 for Syr and 0.9998 for Ole. Additionally, the detection and quantification limits for each analyte were calculated as the concentrations that give a signal-to-noise ratio equal to 3 and 10, respectively. The LODs ranged from 0.005 μg·mL^−1^ for Pin to 7.143 μg·mL^−1^ for Lut, while the LOQs were found between 0.016 and 23.810 μg·mL^−1^, for the same compounds. The linear range was established from LOQ to 250 μg·mL^−1^ in most of the cases, except for the most fluorescent compounds (HTY, TY, Van, and Pin), which saturate the detector and, therefore, did not present such wide linear range (more details in Table 1).

As the LC was connected in series to the FLD detector and to an ESI-IT MS system, the validation of the LC-MS method was carried out simultaneously, calculating analogue parameters to those of the LC-FLD method. Some relevant analytical parameters directly related to the quantification, such as calibration curves equations, determination coefficients, LODs, LOQs and linear dynamic ranges are shown in Table 2. In this way, the analytical performance of the method proposed herein as an alternative to the widely used (for the determination of phenolic compounds from VOOs) LC-MS methodologies could be compared with the analytical figures of merit of a method using MS as detection system. As expected, linear ranges in FLD were wider than in MS detector, except for HTY and Pin which promptly produced signal saturation. In MS detector, LODs and LOQs were generally lower than in FLD, but some exceptions can be found in Table 2.

Since the evaluation of the linear range for compounds whose commercial standard is not available (such as secoiridoid derivatives) could not be performed by using the same approach as the one previously described, it was necessary to use another strategy, which, to a certain extent, could be considered as a third kind of calibration. In this case, the dilution and pre-concentration of QC (between 1% and 200%) was carried out, obtaining eight calibration levels (1%, 10%, 20%, 50%, 75%, 100%, 150% and 200%). Dilution was obviously carried out adding controlled volume of methanol to the QC extract; on the contrary, the pre-concentration was done by evaporating the appropriate amount of QC extract and re-dissolving in the adequate volume. The linearity was then checked by representing the area of the selected compounds (OxHTY, OleAgly (main isomer and isomer 3), DOA, and LigAgly (main peak and its first isomer)) versus the relative concentration of the QC extracts (%). The mentioned peaks were chosen, since they could be properly detected and easily integrated even in the most diluted level. In every case, responses that could be fitted to linear equations were achieved. Indeed, the linear regression equations showed *R^2^* values between 0.9901 and 0.9993 for OleAgly’s main isomer and OxHTY, respectively. The confirmation of this linear behaviour could guarantee that the quantification was being made within a range in which proportional responses for different concentrations of the analytes under evaluation would be obtained; that is a mandatory requirement to assure a correct quantitative determination.

To conclude this section, Supplementary Table S2 provides an overview of some the main aspects (extraction procedure, instrumental platform used, injections needed, number of determined analytes, and FL wavelengths) of the methodology presented in the current contribution, compared with some other LC-FLD methods previously reported in literature. One of the major achievements of the new method is the great number of analytes that can be identified and quantified (26 and 23 phenolic compounds, respectively, belonging to each and every chemical type of the polyphenols found in olive oil). In contrast, 12 is the highest number of phenols which have been quantified with FLD (16 if it is combined with DAD) [5]. The simplicity of the novel method is quite remarkable too; one simple extraction procedure followed by a single injection is needed in this case, whilst the powerful method developed by Godoy-Caballero et al. [5] needs two different protocols for major and minor phenolic compounds extraction (liquid–liquid and SPE, respectively). Moreover, the multi-emission mode allows us to detect each compound, practically, by using the best possible FL conditions, whilst some other previously described methods detect all the phenolic compounds by means of a fixed combination of λ_em_-λ_exc_ or have to use additional injections in different conditions [2].

### 2.4. Analysis of Phenolic Compounds in VOO Samples

Once the developed LC-FLD method was validated, it was applied to quantify 23 phenolic compounds in 10 olive oil samples from different varieties and origins. Both good peak shape and proper resolution were achieved for the compounds under study, as it can be corroborated in Figure 2B, where the chromatograms obtained in the four emission channels for a Picual monovarietal VOO are presented.

Table 3 and Table 4 summarizes the concentrations of the measured phenolic compounds in the selected olive oil samples. To reach our main goal and being able to propose a LC-FLD methodology as a proper and powerful alternative to LC-MS, the quantification of the phenolic compounds whose standard is commercially available was made with both detection systems (see Table 3). For the rest, OxHTY, AcHTY, AcPin and secoiridoid derivatives, the quantitative FL data are presented in Table 4; in those cases, MS data are not included, since these analytes are not quantified in terms of their own pure standard (but by using another standard with a related (but different) structure), and therefore, trying to compare FL and MS results is not a very straightforward task. In other words, we cannot try to establish a proper comparison between the fluorescent behavior and the MS ionization efficiency for compounds with different structures.

The levels of the glycosidic form of Oleuropein (Ole) and some phenolic acids (Gal, Syr, Hmvan, Sin, *m*-Cou and *o*-Cou) were found below the detection limits in all the samples belonging to our sample set (information which was corroborated by the two used detectors (data not shown to contain the size of Table 3)).

Taking into account the data included in Table 3, it is possible to say that the quantitative results from both detectors (FLD and MS) were in good agreement. Thus, no statistically significant differences were found regarding the determined amounts of HTY, TY, 4-HBA, 4-HPA, Van and Fer by both detectors. Blend 2 and Picual were the VOOs showing the highest concentrations of HTY and TY, while, in contrast, Arbequina 1 was the one exhibiting the lowest levels of HTY (0.46 μg·g^−1^) and, Blend 3 and Hojiblanca, the poorest in terms of TY (with 1.2 and 2.0 μg·g^−1^, respectively). 4-HBA was just found in Hojiblanca VOO, with a mean concentration value of 0.29 μg·g^−1^; and 4-HPA could be only determined in Picual oils (1.68 μg·g^−1^). As far as Van in concerned, Blend 3 was the sample presenting the highest amounts of this compound (0.66 μg·g^−1^). Arbequina 2 also exhibited remarkable concentration levels of Van (0.51 μg·g^−1^). Fer was found in six samples (its mean concentration levels varied from 0.058 to 0.166 μg·g^−1^, in Arbequina 1 and Arauco 1, respectively); in three others, it was not quantified; and Blend 3 was the only one in which it not detected.

FL and MS results were also in good agreement regarding, for instance, *p*-Cou. The sample Arauco 1 showed the highest mean levels of *p*-Cou (0.61 μg·g^−1^) and Arbequina 1 the lowest ones (0.11 μg·g^−1^). In two samples (Blends 1 and 3), however, its levels were found under the LOQ with FLD, whereas they could be properly quantified by the MS detector. Something similar was observed for Val, with some of the studied VOOs showing levels below LOD and LOQ with FLD, but properly quantified by MS (which is logical anyway bearing in mind the LOD and LOQ of each detection system given in Table 1). Arbequina 2 (0.51 and 0.52 μg·g^−1^, respectively, in FL and MS) and Blend 3 (0.66 and 0.70 μg·g^−1^, respectively, in FL and MS) were the two examples in which Val was quantified both by FL and MS, achieving statistically equivalent results. Lut could not be quantified in any sample by FL, fact which can be easily understood observing the LOD that this phenolic compound presented (Table 1 and Table 2). Lut’s MS values, however, are given for all of them and were found within the range 1.20–7.0 μg·g^−1^. Hojiblanca VOO was the richest in terms of this flavonoid. The case of Pin deserves to be studied carefully; indeed, in five samples, the quantitative results regarding this lignan, from both FL and MS detectors, showed statistical significant differences (95%; *p* < 0.05). The samples for which contradictory Pin’s results (between FL and MS) were found, presented high contents of DLA (also known as oleocanthal (*m*/*z* 303)) which coeluted with Pin (*m*/*z* 357) and caused ionic suppression in the MS detector. As a consequence, MS detector led to lower Pin´s concentration values than FLD. DLA did not emitted light at 328 nm in FLD, avoiding therefore its presumable interference in that detector and making, from our point of view, more reliable the quantitative value achieved by FLD. In any case, in samples not presenting so remarkably high levels of DLA, MS detector gave results which were in good agreement with those of FL (Aberquina 2 (3.0 μg·g^−1^ in FL and exactly the same value in MS), Arauco 1 (0.95 μg·g^−1^ in FL and 0.87 μg·g^−1^ in MS), Hojiblanca (0.81 μg·g^−1^ in FL and 0.71 μg·g^−1^ in MS), Blend 2 (0.77 μg·g^−1^ in FL and 0.68 μg·g^−1^ in MS) and Blend 3 (3.3 μg·g^−1^ in FL and 2.9 μg·g^−1^ in MS)). Arbequina 2 and Blend 3 were the richest samples in terms of this compound.

With regard to the compounds quantified by using another standard with a related structure (Table 4), it is possible to say that OxHTY was found in all the samples (only in one of them was below the LOQ (Blend 1)), being Picual (2.3 μg·g^−1^) and Blend 2 (1.9 μg·g^−1^) those VOOs with highest concentrations. AcHTY was detected in eight samples and, again, the just mentioned two samples were those exhibiting the maximum concentrations (2.9 and 9.2 μg·g^−1^ in Picual and Blend 2, respectively). OleAgly and its isomers were found in almost all the VOOs; isomer 1 was solely not detected in Changlot and Hojiblanca, isomer 2 was not found in Arbequina 1 and 2, Arauco 1 and Hojiblanca, and isomer 4 was not detected in Blend 2. It seems pertinent to make a comment about the fact of detecting multiple isomers of OleAgly (what is also applicable for LigAgly). This fact has already been discussed by our research group in a recent publication [30], where previous findings regarding the formation of “artificial isomers” of secoiridoids (particularly OleAgly and LigAgly) were corroborated. These isomers show up as long as methanol is used as extractant (usually mixed with water) during the sample preparation. As extensively justified in the cited publication, we think that ignoring these isomers would mean underestimating their “native amount”, which is the reason for finding the five isomers of OleAgly and two of LigAgly (Table 4). With respect to total OleAgly’s concentration, Arauco 2 (274 μg·g^−1^), Blend 1 (187 μg·g^−1^) and Changlot (170 μg·g^−1^) showed considerably high levels when compared to the rest. DOA’s upper levels were determined in Picual (25 μg·g^−1^) and Arbequina 2 (23 μg·g^−1^), while Hojiblanca was the VOO with the minimum concentration of this compound (5.4 μg·g^−1^). As expected, since it is one of the most remarkable features of Arbequina VOOs, one of the Arbequina samples (Arbequina 1) displayed the highest amount of AcPin (11.1 μg·g^−1^). Arbequina 2, Changlot and Blend 1 also exhibited considerable concentrations of this lignan. LigAgly and its two isomers were found in almost all the samples; isomer 2 was the exception, not being found in Arbequina 2 and Blend 3. The three VOOs that were mentioned as the richest in terms of OleAgly were the samples showing most abundant total LigAgly’s content: Arauco 2 (119 μg·g^−1^), Blend 1 (67 μg·g^−1^) and Changlot (54 μg·g^−1^).

Considering the total amount of phenols of each sample (value achieved by adding up all the individual concentrations, just to give an estimation), the sample Arauco 2 was the richest (424 μg·g^−1^), while Hojiblanca was the olive oil with the lowest total levels (40 μg·g^−1^). In addition, Arauco 2 was also the sample exhibiting the largest number of phenolic compounds (a total of 21 phenolic substances (including OleAgly and LigAgly isomers) were found in the mentioned sample).

From the 23 phenolic compounds susceptible to being quantified with the developed method, 16 (including seven secoiridoid isomers) have been found and properly quantified in the selected VOOs.

## 3. Materials and Methods

### 3.1. Reagents and Materials

The reagents used in the present work were of analytical grade and used as received. The solvents used for extraction of the analytes under study from the olive oil samples, methanol and *n*-hexane (both of HPLC grade), were purchased from Panreac (Barcelona, Spain). The mobile phases were prepared with acetonitrile (LC-MS grade) acquired from Lab-Scan (Dublin, Ireland), acetic acid (provided by Panreac) and doubly deionized water obtained using a Milli-Q-system (Millipore, Bedford, MA, USA). Standards of Gal, 4-HBA, 4-HPA, Van, Syr, Hmvan, *o*-, *m*- and *p*- Cou, Sin and Fer, as well as HTY, TY, Lut, Api, Val and DOPAC (IS) were supplied by Sigma-Aldrich (St. Louis, MO, USA), Pin was purchased from Arbo Nova (Turku, Finland), whereas Ole was acquired from Extrasynthese (Lyon, France). The stock solutions were prepared weighing the appropriate amount of each phenolic compound and dissolving it in methanol for obtaining a concentration of 500 µg mL^−1^, and then, they were serially diluted to working concentrations (with concentration levels ranging from 0.5 to 250 µg·mL^−1^). Moreover, different solutions containing previously isolated phenolic compounds [31,32] (AcHTY, EA, LigAgly, OleAgly, DOA, DLA, and AcPin) were used with identification purposes. All the samples and stock solutions were stored in glass coloured flasks at −20 °C and, before the injection into the LC system, both standard solutions and sample extracts were filtered through a Clarinert^TM^ 0.22 μm nylon syringe filter from Agela Technologies (Wilmington, DE, USA).

### 3.2. LC-FLD/MS Analysis

An Agilent 1260-LC system (Agilent Technologies, Waldbronn, Germany) equipped with a vacuum degasser, a binary pump, an autosampler, a DAD and a multiple wavelength FLD was used. Apart from the two mentioned detectors, the chromatographic system was coupled to a Bruker Daltonic Esquire 2000™ ion trap mass spectrometer (Bruker Daltonik, Bremen, Germany) with an ESI interface.

The separation of the target compounds was performed using a Zorbax Eclipse Plus C_18_ analytical column (4.6 × 150 mm, 1.8 μm particle size) protected by a guard cartridge of the same packing, operating at room temperature and a flow rate of 0.8 mL·min^−1^. The mobile phases used were water with acetic acid (0.5%) (Phase A) and acetonitrile (Phase B), and the solvent gradient changed according to the following conditions [7]: 0 to 10 min, 5%–30% B; 10 to 12 min, 30%–33% B; 12 to 17 min, 33%–38% B; 17 to 20 min, 38%–50% B; 20 to 23 min, 50%–95% B. Finally, the B content was decreased to the initial conditions (5%) in 2 min and the column re-equilibrated for 2.5 min. A volume of 10 μL of the methanolic extracts of olive oil, pure or isolated standards and standard mixtures was injected in each case.

The separated compounds were monitored on-line with the FLD, DAD, and the ESI-IT MS detectors. In the first one, excitation wavelength was set at 285 nm and four channels with the following emission wavelengths λ: 316 nm, 328 nm, 350 nm, and 450 nm, were used in order to obtain four different chromatographic traces per run. Some other important parameters in that detector were 2.31 Hz for signal acquisition rate (0.2 min of peak width which corresponds to a response time of 4 s), 10 units for photomultiplier (PMT) gain, 5% of Zero offset and 100 Lu of attenuation in analog output. Besides, previously optimized conditions for phenolic compounds detection were employed in DAD (240, 280, and 330 nm) (data has not been considered in the manuscript to contain the size of the contribution) and the MS detector [7]. The most relevant ionization source and transferring MS parameters resulted to be: nebulizer pressure, drying gas flow and drying temperature, which were set at 30 psi, 9 L·min^−1^ and 300 °C, respectively; and voltages in the end plate offset and in the capillary, which were −500 V and +3200 V, apiece.

### 3.3. Samples and Sample Preparation

The VOO samples studied in this work were commercial samples, acquired from Argetinean, Spanish and Moroccan factories. The samples included monovarietal olive oils from different varieties: two Arbequina (one Spanish (number 1 in Table 2) and another Argentinean (number 2)), two Arauco (both Argentinean), one Changlot (Argentinean), one Hojiblanca (Spanish), one Picual (Spanish); and three blends (two Moroccan (numbers 1 and 2) and another Spanish (number 3)). The oils were extracted from olive fruits with maturity index around 3 by a two-phase continuous centrifugation process. All the samples were kept refrigerated in their original containers until their analysis. With the just described sample selection, we logically attempted to demonstrate the potential of our method by using accessible olive oils coming from different countries rather than carrying out an agronomical comparison among the selected samples.

The phenolic compounds were isolated by using a liquid–liquid extraction according to a previously reported procedure [33], which can be briefly described as follows: 2.0 ± 0.1 g of olive oil were weighed in a test tube with a screw cap and 0.025 mL of IS solution (at a concentration of 500 mg·L^−1^) were added. After solvent evaporation of the IS (using nitrogen), 1 mL of n-hexane was added and the tube was shaken in a vortex. Then, the compounds of interest were extracted three times, by adding 2 mL of methanol/water (60:40, *v/v*), shaking during 2 min and centrifuging at 3500 rpm for 6 min (each time). The supernatants were put together and evaporated to dryness using a rotary evaporator. The residue was finally redissolved in 1 mL of methanol and filtered through a 0.22 μm membrane filter.

A QC sample was used to assure the stability of the system and to evaluate different analytical parameters. This QC sample was made by mixing equivalent volumes of the extracts of each variety of the selected VOOs. For validation purposes, the mentioned QC sample and the standard mix composed by the 18 compounds previously mentioned in Section 3.1 were used.

## 4. Conclusions

In the present work, a LC-FLD methodology was developed for the determination of phenolic compounds in olive oil. The deep evaluation of the fluorescence features of the analytes, the correct selection of emission/excitation wavelengths, and the selection of the most appropriate fluorescence mode (multi-channels) were pivotal steps in the optimization of the methodology. Validation studies were carried out, paying particular attention to precision, trueness and possible matrix effects and the results were very satisfactory. With the final goal of comparing the FL and MS quantitative results, analytical figures of merit were also determined for MS. The applicability of the LC-FLD method was finally evaluated carrying out the analysis of ten olive oil samples (different origins and varieties), and comparing (when possible) the results achieved by FL with those of MS.

The presented methodology allowed the selective determination of a considerable number of compounds (23 (plus seven isomers) which could be quantified and 26 (plus isomers), also counting those three that have been reported for the first time using FLD). It has been demonstrated that FLD is an affordable and powerful tool for the determination of these relevant minor components of the olive oil.

It is imperative to continue working in this direction, searching for simple, repeatable, reliable, affordable and easily adaptable methodologies to be used in routine analytical labs. However, the analytical part is not the only “piece of the puzzle” which has to be addressed, it is also necessary to clarify the terminology in this context and get a consensus among scientists regarding the formulation of the health claim on “olive oil polyphenols” (with regard to wording and even the specified level in the conditions of use of the claim (which absolutely depends on the quantitative approach used)).

## Figures and Tables

**Table 1:** Analytical parameters related to the evaluation of accuracy and matrix effect of the developed LC-FLD method.

Compound	Accuracy					Matrix Effect		
	*Intra-*Day Repeatability (% RSD) ^a^		*Inter-*Day Repeatability (% RSD) ^b^		Trueness (%) ^c^	Solvent Calibration Slope	Matrix Calibration Slope	Matrix Slope/Solvent Slope
	*Area*	*t_r_*	*Area*	*t_r_*				
Gal	2.72	0.39	5.70	0.41	92.83	0.127	0.137	1.08
HTY	6.80	0.34	6.91	0.40	103.66	10.226	9.041	0.88
TY	2.17	0.26	3.40	0.47	90.08	6.239	5.257	0.86
4-HBA	3.34	0.17	4.27	0.46	101.98	0.880	0.983	1.12
4-HPA	3.68	0.19	4.66	0.50	85.49	2.012	2.104	1.05
Van	3.68	0.21	3.74	0.51	101.01	14.276	13.384	0.94
Syr	3.49	0.25	4.80	0.54	104.71	6.770	6.748	1.00
Hmvan	9.05	0.24	11.98	0.56	87.53	1.244	1.273	1.02
*p*-Cou	2.94	0.18	3.51	0.58	86.03	0.272	0.285	1.05
Val	2.94	0.23	4.42	0.53	85.28	0.299	0.323	1.08
Sin	3.75	0.26	4.38	0.63	80.55	0.618	0.678	1.10
Fer	2.35	0.24	2.79	0.61	88.06	1.287	1.410	1.10
*m*-Cou	3.33	0.21	5.93	0.63	111.81	0.083	0.087	1.05
Ole	4.32	0.34	6.83	0.81	112.28	1.246	1.411	1.13
*o*-Cou	2.52	0.24	2.92	0.70	84.27	1.170	1.288	1.10
Lut	*	*	*	*	100.74	0.009	0.011	1.16
Pin	2.35	0.36	2.13	0.99	100.46	40.452	44.258	1.09

^a^ RSD values (%) for peak areas or retention times of the analytes under study measured from five injections of the fortified QC carried out within the same sequence; ^b^ RSD values (%) for peak areas or retention times of the analytes under study measured from five injections of the fortified QC (belonging to five different sequences carried out over five days); * Lut was found under the detection limits in the fortified QC, so its repeatability could not be measured; ^c^ Trueness was measured by calculating the recovery (%), and it was estimated by analyzing the same sample extracted before and after the standard addition and calculating the difference between the obtained results. The values included in this table are those achieved for the intermediate concentration level (except for Lut, whose values belong to a higher concentration level).

**Table 2:** Comparison between calibration functions, determination coefficients, LODs, LOQs and lineal dynamic ranges for the FLD and the MS detectors.

Analyte	Detector	External Calibration Curve	*R^2^*	LOD (μg·mL^−1^)	LOQ (μg·mL^−1^)	Linear Range (μg·mL^−1^) ^a^
Gal	FLD	*y* = 0.127*x* + 0.1263	0.9987	0.625	2.083	250
	MS	*y* = 40519*x* + 37647	0.9965	0.051	0.171	100
HTY	FLD	*y* = 10.226*x* + 2.1124	0.9989	0.035	0.115	20
	MS	*y* = 52263*x* + 55720	0.9945	0.017	0.057	50
TY	FLD	*y* = 6.239*x* + 3.3395	0.9993	0.009	0.029	100
	MS	*y* = 20379*x* + 17714	0.9924	0.058	0.195	50
4-HBA	FLD	*y* = 0.880*x* + 2.804	0.9954	0.036	0.121	250
	MS	*y* = 16457*x* + 49330	0.9911	0.061	0.204	150
4-HPA	FLD	*y* = 2.012*x* + 2.6778	0.9982	0.357	1.190	250
	MS	*y* = 13242*x* − 2729.7	0.9938	0.122	0.407	150
Van	FLD	*y* = 14.276*x* − 0.4684	0.9989	0.004	0.013	20
	MS	*y* = 16919*x* + 11618	0.9956	0.025	0.084	10
Syr	FLD	*y* = 6.770*x* + 8.7496	0.9861	0.007	0.023	50
	MS	*y* = 27088*x* + 14618	0.9928	0.025	0.083	10
Hmvan	FLD	*y* = 1.244*x* + 0.4454	0.9981	0.556	1.852	250
	MS	*y* = 31576*x* − 42195	0.9983	0.155	0.515	50
*p*-Cou	FLD	*y* = 0.272*x* + 1.036	0.9932	0.031	0.103	250
	MS	*y* = 40281*x* + 21180	0.9941	0.018	0.059	20
Val	FLD	*y* = 0.299*x* + 0.631	0.9974	0.090	0.301	250
	MS	*y* = 12440*x* + 7246.2	0.9927	0.055	0.182	10
Sin	FLD	*y* = 0.618*x* + 0.8065	0.9986	0.039	0.132	250
	MS	*y* = 58259*x* + 28312	0.9939	0.021	0.069	10
Fer	FLD	*y* = 1.287*x* + 3.1028	0.9965	0.021	0.069	250
	MS	*y* = 39342*x* + 13674	0.9949	0.022	0.073	10
*m*-Cou	FLD	*y* = 0.083*x* + 0.122	0.9995	0.306	1.020	250
	MS	*y* = 58990*x* + 130287	0.9934	0.013	0.043	100
Ole	FLD	*y* = 1.246*x* + 0.1575	0.9998	0.273	0.909	250
	MS	*y* = 6709*x* + 86	0.9990	0.050	0.165	20
*o*-Cou	FLD	*y* = 1.170*x* + 3.713	0.9954	0.022	0.072	250
	MS	*y* = 33291*x* + 12614	0.9946	0.077	0.255	100
Lut	FLD	*y* = 0.009*x* + 0.0064	0.9984	7.143	23.810	250
	MS	*y* = 201100*x* + 44947	0.9994	0.005	0.016	10
Pin	FLD	*y* = 40.452*x* + 7.795	0.9991	0.005	0.016	10
	MS	*y* = 34634*x* + 63107	0.9912	0.032	0.108	50

^a^ Linear ranges were established from LOQ to the indicated value.

**Table 3:** Content of phenolic compounds with available commercial pure standard (expressed in μg·g^−1^) determined by FL and MS detectors coupled to LC.

Compound	Detector	Arbequina 1	Arbequina 2	Arauco 1	Arauco 2	Changlot	Hojiblanca	Picual	Blend 1	Blend 2	Blend 3
HTY	FLD	0.46 ± 0.03	5.0 ± 0.3	5.8 ± 0.4	4.3 ± 0.3	5.0 ± 0.3	0.66 ± 0.04	11.2 ± 0.8	3.0 ± 0.2	19.2 ± 1.2	1.6 ± 0.1
	MS	0.48 ± 0.03	4.8 ± 0.3	6.3 ± 0.4	4.2 ± 0.3	5.0 ± 0.3	0.74 ± 0.05	11.4 ± 0.5	2.8 ± 0.2	17 ± 1	1.5 ± 0.1
TY	FLD	2.8 ± 0.2	4.9 ± 0.3	13.9 ± 0.8	11.7 ± 0.8	6.8 ± 0.3	2.0 ± 0.1	14.5 ± 0.7	15.3 ± 0.9	24 ± 1	1.2 ± 0.1
	MS	3.1 ± 0.2	4.7 ± 0.2	15 ± 1	10.2 ± 0.7	6.2 ± 0.3	2.3 ± 0.2	15.8 ± 0.7	14.8 ± 0.6	24 ± 1	1.2 ± 0.1
4-HBA	FLD	n.d.	n.d.	n.d.	n.d.	n.d.	0.29 ± 0.01	n.d.	n.d.	n.d.	n.d.
	MS	n.d.	n.d.	n.d.	n.d.	n.d.	0.31 ± 0.01	n.d.	n.d.	n.d.	n.d.
4-HPA	FLD	n.d.	n.d.	n.d.	n.d.	n.d.	n.d.	1.68 ± 0.07	n.d.	n.d.	n.d.
	MS	n.d.	n.d.	n.d.	n.d.	n.d.	n.d.	1.72 ± 0.07	n.d.	n.d.	n.d.
Van	FLD	0.29 ± 0.02	0.45 ± 0.03	0.91 ± 0.06	0.39 ± 0.02	n.q.	0.43 ± 0.02	0.76 ± 0.04	2.0 ± 0.1	0.61 ± 0.02	n.d.
	MS	0.26 ± 0.02	0.41 ± 0.02	0.82 ± 0.04	0.43 ± 0.02	n.d.	0.40 ± 0.02	0.68 ± 0.04	1.8 ± 0.1	0.58 ± 0.03	n.d.
*p*-Cou	FLD	0.11 ± 0.01	0.21 ± 0.01	0.61 ± 0.04	0.32 ± 0.02	0.25 ± 0.01	0.28 ± 0.02	0.38 ± 0.02	n.q.	0.15 ± 0.01	n.q.
	MS	0.13 ± 0.01	0.21 ± 0.01	0.57 ± 0.03	0.35 ± 0.02	0.25 ± 0.01	0.25 ± 0.02	0.36 ± 0.02	0.061 ± 0.003	0.17 ± 0.01	0.093 ± 0.005
Val	FLD	n.q.	0.51 ± 0.02	n.q.	n.q.	n.q.	n.q.	n.d.	n.q.	n.q.	0.66 ± 0.03
	MS	0.134 ± 0.007	0.52 ± 0.03	0.102 ± 0.004	0.113 ± 0.006	0.135 ± 0.007	n.q.	n.q.	n.q.	0.143 ± 0.008	0.70 ± 0.03
Fer	FLD	0.058 ± 0.002	0.081 ± 0.004	0.166 ± 0.005	0.076 ± 0.002	0.126 ± 0.005	n.q.	0.071 ± 0.002	n.q.	n.q.	n.d.
	MS	0.055 ± 0.002	0.080 ± 0.004	0.176 ± 0.006	0.074 ± 0.002	0.122 ± 0.005	n.q.	0.069 ± 0.002	n.q.	n.q.	n.d.
Lut	FLD	n.q.	n.q.	n.q.	n.q.	n.d.	n.q.	n.d.	n.d.	n.d.	n.d.
	MS	5.7 ± 0.3	4.4 ± 0.1	6.2 ± 0.3	5.3 ± 0.3	2.6 ± 0.1	7.0 ± 0.4	3.4 ± 0.2	1.22 ± 0.07	1.20 ± 0.06	3.0 ± 0.1
Pin	FLD	2.09 ± 0.09 *	3.0 ± 0.1	0.95 ± 0.04	0.90 ± 0.04 *	2.7 ± 0.1 *	0.81 ± 0.05	4.0 ± 0.2 *	3.1 ± 0.1 *	0.77 ± 0.05	3.3 ± 0.2
	MS	0.91 ± 0.05 *	3.0 ± 0.1	0.87 ± 0.05	0.39 ± 0.02 *	1.6 ± 0.1 *	0.71 ± 0.05	2.4 ± 0.1 *	1.3 ± 0.1 *	0.68 ± 0.05	2.9 ± 0.2

Every result is the average of three independent (sample preparation and injection) determinations (*n* = 3). The results are given by the mean value ± SD. n.d.: non detected; n.q.: non quantified; * Quantitative results from both FL and MS detectors have statistical significant differences (95%; *p* < 0.05).

**Table 4:** Content of phenolic compounds whose pure standard is not commercially available (expressed in μg·g^−1^) achieved by LC-FLD.

Compound		Arbequina 1	Arbequina 2	Arauco 1	Arauco 2	Changlot	Hojiblanca	Picual	Blend 1	Blend 2	Blend 3
OxHTY		0.163 ± 0.008	1.87 ± 0.09	1.91 ± 0.09	0.19 ± 0.01	0.34 ± 0.02	0.122 ± 0.006	2.3 ± 0.1	n.q.	1.9 ± 0.1	0.51 ± 0.02
AcHTY		1.34 ± 0.07	0.47 ± 0.02	1.10 ± 0.06	0.41 ± 0.02	n.d.	0.27 ± 0.01	2.9 ± 0.2	n.d.	9.2 ± 0.5	0.20 ± 0.01
OleAgly	Isomer 1	0.74 ± 0.04	1.04 ± 0.05	2.7 ± 0.1	5.4 ± 0.3	n.d.	n.d.	n.q.	10.8 ± 0.5	1.10 ± 0.06	6.1 ± 0.3
	Isomer 2	n.d.	n.d.	n.d.	25 ± 1	19.2 ± 0.9	n.d.	15.8 ± 0.6	115 ± 6	48 ± 2	43 ± 2
	Isomer 3	1.50 ± 0.07	2.6 ± 0.1	11.7 ± 0.6	85 ± 4	58 ± 3	2.1 ± 0.1	n.q.	7.8 ± 0.4	13.3 ± 0.6	3.4 ± 0.2
	Isomer 4	2.0 ± 0.1	2.4 ± 0.2	5.5 ± 0.3	8.9 ± 0.5	3.3 ± 0.2	1.46 ± 0.07	12.7 ± 0.8	10.1 ± 0.5	n.d.	3.9 ± 0.2
	Main isomer	12.7 ± 0.6	7.5 ± 0.4	32 ± 2	147 ± 7	87 ± 4	6.5 ± 0.3	36 ± 2	24 ± 1	28 ± 1	11.1 ± 0.6
	Isomer 5	4.1 ± 0.2	3.1 ± 0.1	1.16 ± 0.04	3.1 ± 0.1	2.5 ± 0.1	2.6 ± 0.1	4.3 ± 0.2	19.1 ± 0.8	3.7 ± 0.2	3.8 ± 0.1
	Total	21.1 ± 0.6	16.6 ± 0.4	53 ± 2	274 ± 8	170 ± 5	12.8 ± 0.3	69 ± 2	187 ± 6	94 ± 1	72 ± 2
DOA		12.1 ± 0.6	23 ± 1	11.5 ± 0.6	11.8 ± 0.6	16.6 ± 0.9	5.4 ± 0.2	25 ± 1	18.4 ± 0.9	10.0 ± 0.4	11.6 ± 0.6
AcPin		11.1 ± 0.6	2.5 ± 0.1	1.21 ± 0.06	1.02 ± 0.05	3.2 ± 0.1	0.82 ± 0.05	1.08 ± 0.05	3.2 ± 0.1	1.52 ± 0.08	2.14 ± 0.09
LigAgly	Isomer 1	10.6 ± 0.6	2.8 ± 0.2	10.8 ± 0.6	103 ± 6	38 ± 2	5.9 ± 0.4	17 ± 1	19 ± 1	7.1 ± 0.4	4.7 ± 0.2
	Main isomer	7.6 ± 0.4	18.7 ± 0.9	9.6 ± 0.5	11.3 ± 0.6	10.3 ± 0.5	4.4 ± 0.2	18.8 ± 0.9	36 ± 2	6.4 ± 0.3	16.2 ± 0.8
	Isomer 2	6.7 ± 0.3	n.d.	5.9 ± 0.3	5.1 ± 0.3	6.1 ± 0.3	5.8 ± 0.3	8.4 ± 0.4	11.4 ± 0.5	16.1 ± 0.8	n.d.
	Total	24.8 ± 0.8	21.4 ± 0.9	26.2 ± 0.8	119 ± 6	54 ± 2	16.1 ± 0.5	44 ± 1	67 ± 2	30 ± 1	20.9 ± 0.8

Every result is the average of three independent (sample preparation and injection) determinations (*n* = 3). The results are given by the mean value ± SD. OxHTY and AcHTY were quantified in terms of HTY; AcPin, by using the calibration curve of Pin; OleAgly and its isomers, DOA, and LigAgly and its two isomers were quantified in terms of Ole. n.d.: non detected; n.q.: non quantified.

## Supplementary Materials

Supplementary materials can be found at www.mdpi.com/1422-0067/17/10/1627/s1.
