# Supplementary material for: Potential of LC Coupled to Fluorescence Detection in Food Metabolomics: Determination of Phenolic Compounds in Virgin Olive Oil
Source: Int J Mol Sci. 2016 Sep 24;17(10):1627. doi: 10.3390/ijms17101627 (PMC5085660; doi:10.3390/ijms17101627)
Supplement: Supplementary file 1 [file ijms-17-01627-s001.pdf]

Supplementary Materials: Potential of LC Coupled to Fluorescence Detection in Food Metabolomics: Determination of Phenolic Compounds in Virgin Olive Oil

Romina P. Monasterio, Lucía Olmo-García, Aadil Bajoub, Alberto Fernández-Gutiérrez and Alegría Carrasco-Pancorbo

Table S1. Molecular formula, chemical structure, retention time and fluorescence maxima of the phenolic compounds under study.

Compounds	t_R (min)	Molecular Formula	Structure	λ_{exc} (nm)	λ_{em} (nm)	Selected λ_{em} (nm)	Group
Oxidized hydroxytyrosol (OxHTY)	3.3	$C_8H_8O_3$	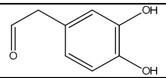	235, **285**	324	328	Simple phenolic alcohols
Gallic acid (Gal)	4.8	$C_7H_6O_5$	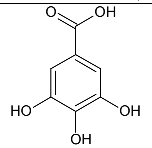	278 [a]	376	350	Benzoic derivate
Hydroxytyrosol (HTY)	6.9	$C_8H_{10}O_3$	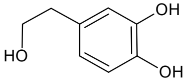	235, **285**	324	328	Simple phenolic alcohols
Tyrosol (TY)	8.6	$C_8H_{10}O_2$	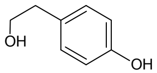	232, **283**	313	316	Simple phenolic alcohols
4-Hydroxybenzoic acid (4-HBA)	9.0	$C_7H_6O_3$	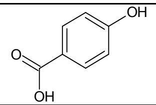	265	328, **340**	350	Benzoic derivate
4-Hydroxyphenylacetic acid (4-HPA)	9.4	$C_8H_8O_3$	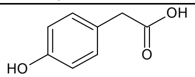	234, **280**	316	316	Other phenolic compounds
Vanillic acid (Van)	9.7	$C_8H_8O_4$	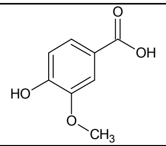	**269**, 294	356	350	Benzoic derivate

Table S1. *Cont.*

Compounds	t_R (min)	Molecular Formula	Structure	λ_{exc} (nm)	λ_{em} (nm)	Selected λ_{em} (nm)	Group
Syringic acid (Syr)	9.8	$C_9H_{10}O_5$	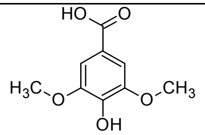	233, **285**	362	350	Benzoic derivate
Homovanillic acid (Hmvan)	10.0	$C_9H_{10}O_4$	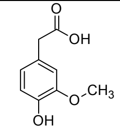	239, **283**, 298 [a]	320	316	Benzoic derivate
p-coumaric acid (*p*-Cou)	11.6	$C_9H_8O_3$	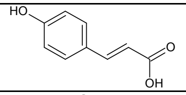	239 [b], **300** [b]	414	450	Hydroxycinnamic derivate
Vanillin (Val)	11.8	$C_8H_8O_3$	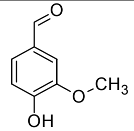	241 [b], **296** [b]	415	450	Other phenolic compounds
Sinapic acid (Sin)	12.1	$C_{11}H_{12}O_5$	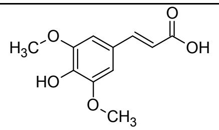	249 [b], **310** [b]	448	450	Hydroxycinnamic derivate
Ferulic acid (Fer)	12.3	$C_{10}H_{10}O_4$	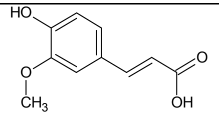	250 [b], **303** [b]	440	450	Hydroxycinnamic derivate
m-coumaric acid (*m*-Cou)	12.8	$C_9H_8O_3$	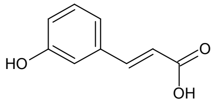	285 [b]	434	450	Hydroxycinnamic derivate

Table S1. *Cont.*

Compounds	t_R (min)	Molecular Formula	Structure	λ_{exc} (nm)	λ_{em} (nm)	Selected λ_{em} (nm)	Group
Hydroxytyrosol acetate (AcHTY)	13.2	$C_9H_{10}O_4$	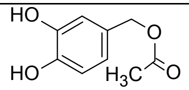	235, **285**	323	328	Simple phenolic alcohols
Oleuropein (Ole)	13.6	$C_{25}H_{32}O_{13}$	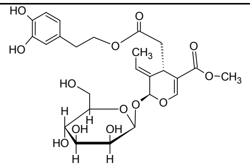	236, **285**, 328 [b]	324	328	Secoiridoids
o-coumaric acid (*o*-Cou)	14.0	$C_9H_8O_3$	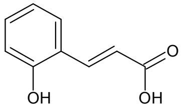	**284** [b], 322 [b]	**454**, 522	450	Hydroxycinnamic derivate
Oleuropein aglycone (OleAgly)	15.2, 18.5, 19.5, 19.8, 21.5 (main isomer), 22.2	$C_{19}H_{22}O_8$	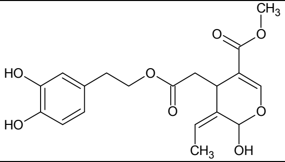	235, **285**	321	316	Secoiridoids
Luteolin (Lut)	16.5	$C_{15}H_{10}O_6$	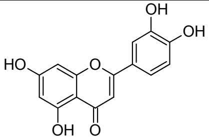	255	330, **434**	450	Flavonoids
Decarboxymethyl oleuropein aglycone (DOA)	16.7	$C_{18}H_{20}O_4$	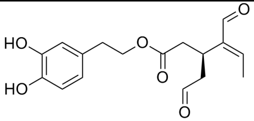	240, **284**	316	316	Secoiridoids

Table S1. *Cont.*

Compounds	t_R (min)	Molecular Formula	Structure	λ_{exc} (nm)	λ_{em} (nm)	Selected λ_{em} (nm)	Group
Pinoresinol (Pin)	17.3	$C_{20}H_{22}O_6$	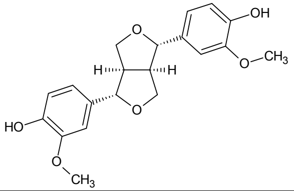	238, **285**	323	328	Lignans
Acetoxypinoresinol (AcPin)	17.9	$C_{22}H_{24}O_8$	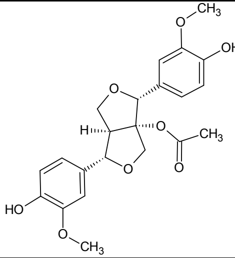	240, **284**	322	328	Lignans
Ligtroside Aglycone (LigAgly)	22.4, 23.8 (main isomer), 24.0	$C_{19}H_{22}O_7$	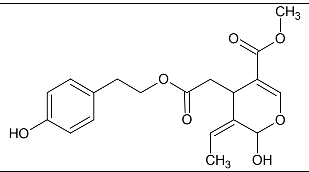	232, **281**	314	316	Secoiridoids

Maximum excitation wavelengths obtained when λ_{em} is set at 325 nm except for [a] (360 nm) and [b] (450 nm). Maximum emission wavelengths obtained when λ_{exc} is set at 285 nm. In both columns of λexc and λem (nm), we highlight in bold letter the most convenient wavelength value when more than one fluorescence maximum were found.

Table S2. Comparison between the methodology described herein and the previously published methods with FL detection for the determination of phenolic compounds in olive oil and related matrixes.

Analytical Technique	Extraction Procedure of the Phenolic Compounds	Matrix	Number of Needed Injections in FLD	Number of Analytes Using FLD/ Total Analytes	Purpose	FLD Wavelengths (nm) for Each Analyte	Ref.
LC-DAD-FLD/LC-MS	SPE (1 g olive powder → 5 mL methanol/water (50:50 *v*/*v*), dilution 1:10)	Olives	2	6/26	Identification	λ_{exc} = 280, λ_{em} = 320 (TY, Van, Ole); λ_{em} = 320 (chlorogenic acid, caffeic acid (Caf), *p*-Cou)	[17]
LC-DAD-FLD	LLE (0.6 mL olive oil → 1.8 mL DMF)	Olive oil	1	9/14	Quantification of lignans and identification of the rest	λ_{exc} = 280, λ_{em} = 320 (HTY, TY, AcHTY, AcPin, Pin, OleAgly, LigAgly, DOA, decarboxymethyl ligstroside aglycon (DLA))	[15]
LC-DAD-FLD	LLE (0.6 mL olive oil → 1.8 mL DMF)	Olive oil	1	9/14	Identification	λ_{exc} = 280, λ_{em} = 320 (HTY, TY, AcHTY, AcPin, Pin, OleAgly, LigAgly, DOA, DLA)	[16]
LC-FLD	LLE (50 µL rat plasma → 100 µL water)	Rat plasma	1	2	Quantification	λ_{exc} = 281, λ_{em} = 316 (HTY, Ole)	[21]
LC-DAD-FLD	LLE (90g olive oil → 10 mL); Direct injection (2 g olive oil + 10 mL acetone)	Olive oil	1	7/7	Quantification	λ_{exc} = 280, λ_{em} =353 (HTY, TY, OleAgly, DOA); λ_{em} = 313 (DLA); λ_{em} = 339 (Pin, AcPin)	[22]
CE-DAD-FLD	LLE (5 g olive oil → 1 mL ethanol); Direct injection for HTY, Van, Caf (6 mL olive oil + 6 mL 1-propanol)	Olive oil	1	5/9	Quantification	λ_{exc} = 297, λ_{em} = 320 (gentisic acid (Gen), Caf, Van, HTY, *o*-Cou)	[20]
CE-DAD-FLD	Direct injection for HTY and Van (6 mL olive oil + 6 mL 1-propanol); SPE (60 g olive oil → 2 mL methanol)	Olive oil	1	6/9	Quantification	λ_{exc} = 297, λ_{em} = 320 (quercetin, Gen, Caf, Van, HTY, *o*-Cou)	[18]
LC-DAD-FLD	LLE for HTY and TY (1g → 2 mL ethanol, dilution 1:10); SPE for the rest (15 g → 1 mL methanol/water (50:50 *v*/*v*), dilution 1:2)	Olive oil	2	12/16	Quantification	λ_{exc} = 300, λ_{em} = 330 (4-HPA, 4-HBA); λ_{em} = 350 (Van, HTY and TY); λ_{em} = 380 (Syr); λ_{em} = 450 (Gal, Gen, Fer, *p*-Cou, *o*-Cou)	[19]
LC-DAD-FLD	LLE (3 g olive oil → 4 mL methanol/water (60:40 *v*/*v*))	Olive oil	1	4/8	Quantification	λ_{exc} = 250, λ_{em} = 350 (HTY, TY, Van, OleAgly)	[23]
LC-DAD-FLD	LLE (3 g olive oil → 4 mL methanol/water (60:40 *v*/*v*))	Olive oil	1	7/11	Quantification	λ_{exc} = 250, λ_{em} = 350 (HTY, TY, Van, OleAgly, LigAgly, DOA, DLA)	[24]
LC-FLD	LLE (2 g olive oil → 1 mL methanol)	Olive oil	1	26 (plus 7 isomers)	Quantification of 23 of them (plus 7 isomers)	See Table S1	This methodology
